# Supplementary material for: Distinguishing different modes of growth using single-cell data
Source: eLife. 2021 Dec 2;10:e72565. doi: 10.7554/eLife.72565 (PMC8727026; doi:10.7554/eLife.72565)
Supplement: Supplementary file 1. [file elife-72565-supp1.pdf]

Distinguishing different modes of growth using single-cell data - Supplementary Information

Prathitha Kar[1,2], Sriram Tiruvadi-Krishnan[3], Jaana Männik[3], Jaan Männik*[3], and Ariel Amir†[1]

[1]*School of Engineering and Applied Sciences, Harvard University, Cambridge, MA 02134, USA*

[2]*Department of Chemistry and Chemical Biology, Harvard University, Cambridge, MA 02138, USA*

[3]*Department of Physics and Astronomy, University of Tennessee, Knoxville, TN 37996, USA*

*Corresponding author- email: jmannik@utk.edu; phone: +1 (865) 974 6018

†Corresponding author- email: arielamir@seas.harvard.edu; phone: +1 (617) 495 5818

Table S1: Variable definitions.

**Variables**	**Description**
L_b	Length of the cell at birth and also a proxy for size at birth
L_d	Length of the cell at division and also a proxy for size at division
l_b	$\frac{L_b}{\langle L_b \rangle}$, where $\langle L_b \rangle$ is mean size at birth
l_d	$\frac{L_d}{\langle L_b \rangle}$, where $\langle L_b \rangle$ is mean size at birth
$\mathrm{f}(l_b)$	Mathematical function which captures the regulation strategy determining division given size at birth. $\mathrm{f}(l_b) = 2l_b^{1-\alpha}$
T_d	Generation time
σ_t	Standard deviation of generation time
x_n or x	$x_n = \ln(l_b^n)$. Since $l_b \approx 1$, $x_n \approx l_b^n - 1$
σ_x	Standard deviation of x_n
$f_1(x_n)$	Gaussian describing the distribution of x_n. $f_1(x_n) = \frac{1}{\sqrt{2\pi\sigma_x^2}} \exp\left(-\frac{x_n^2}{2\sigma_x^2}\right)$
$\langle \lambda \rangle$	Mean growth rate
CV_λ	Coefficient of variation of growth rate
$\xi(0, CV_\lambda)$	Normally distributed growth rate noise. Growth rate is defined as $\lambda = \langle \lambda \rangle + \langle \lambda \rangle \xi(0, CV_\lambda)$
$f_2(\xi)$	Gaussian describing the distribution of random variable $\xi(0, CV_\lambda)$. $f_2(\xi) = \frac{1}{\sqrt{2\pi CV_\lambda^2}} \exp\left(-\frac{\xi^2}{2CV_\lambda^2}\right)$
$\frac{\zeta(0,\sigma_n)}{\langle \lambda \rangle}$	Normally distributed time additive division timing noise with mean 0 and standard deviation $\frac{\sigma_n}{\langle \lambda \rangle}$

$f_3(\zeta)$	Gaussian describing the distribution of random variable $\zeta(0, \sigma_n)$. $f_3(\zeta) = \frac{1}{\sqrt{2\pi\sigma_n^2}} \exp\left(-\frac{\zeta^2}{2\sigma_n^2}\right)$
$\zeta_s(0, \sigma_{bd})$	Normally distributed size additive division timing noise with mean 0 and standard deviation σ_{bd}
σ_l	Standard deviation of $\ln(\frac{L_d}{L_b})$
$f_4\left(\ln(\frac{L_d}{L_b})\right)$	Gaussian describing the distribution of $\ln(\frac{L_d}{L_b})$. $f_4\left(\ln(\frac{L_d}{L_b})\right) = \frac{1}{\sqrt{2\pi\sigma_l^2}} \exp\left(-\frac{\left(\ln(\frac{L_d}{L_b})-\ln(2)\right)^2}{2\sigma_l^2}\right)$
ρ_{exp}	Correlation coefficient of the pair $(\ln(\frac{L_d}{L_b}), \langle\lambda\rangle T_d)$
m_{tl}	Slope of the best linear fit for $\ln(\frac{L_d}{L_b})$ vs $\langle\lambda\rangle T_d$ plot
c_{tl}	Intercept of the best linear fit for $\ln(\frac{L_d}{L_b})$ vs $\langle\lambda\rangle T_d$ plot
m_{lt}	Slope of the best linear fit for $\langle\lambda\rangle T_d$ vs $\ln(\frac{L_d}{L_b})$ plot
c_{lt}	Intercept of the best linear fit for $\langle\lambda\rangle T_d$ vs $\ln(\frac{L_d}{L_b})$ plot
$\langle\lambda_{lin}\rangle$	Mean normalized elongation speed
$CV_{\lambda,lin}$	Coefficient of variation of normalized elongation speed
$\xi_{lin}(0, CV_{\lambda,lin})$	Normally distributed normalized elongation speed noise. Normalized elongation speed is defined as $\lambda_{lin} = \langle\lambda_{lin}\rangle + \langle\lambda_{lin}\rangle\xi_{lin}(0, CV_{\lambda,lin})$
$\sigma_{l,lin}$	Standard deviation of $l_d - l_b$
ρ_{lin}	Correlation coefficient of the pair $(l_d - l_b, \langle\lambda_{lin}\rangle T_d)$
$m_{tl,lin}$	Slope of the best linear fit for $l_d - l_b$ vs $\langle\lambda_{lin}\rangle T_d$ plot
$c_{tl,lin}$	Intercept of the best linear fit for $l_d - l_b$ vs $\langle\lambda_{lin}\rangle T_d$ plot
$m_{lt,lin}$	Slope of the best linear fit for $\langle\lambda_{lin}\rangle T_d$ vs $l_d - l_b$ plot
$c_{lt,lin}$	Intercept of the best linear fit for $\langle\lambda_{lin}\rangle T_d$ vs $l_d - l_b$ plot
L_i	Cell size at the start of DNA replication (initiation)

$L_i^{tot,next}$	Total cell size of the daughter cells at the start of DNA replication
Δ_{ii}	Size added per origin between initiations
O	Number of origins just after initiation
C+D	Time between initiation and division
T_n	Timing of start of septum formation/onset of constriction
L_n	Cell size at time T_n

Table S2: The slope and the intercept of the best linear fit along with their 95% confidence intervals (CI) obtained on performing linear regression on experimental data. The data is collected for cells growing in M9 alanine, glycerol and glucose-cas media [S1].

**Media**	**No. of cells**	$\mathbf{T_d}$ **(min)**	$\ln(\frac{L_d}{L_b})$ **vs** $\langle\lambda\rangle T_d$ **plot**		$\langle\lambda\rangle T_d$ **vs** $\ln(\frac{L_d}{L_b})$ **plot**	
			Slope (with 95% CI)	Intercept (with 95% CI)	Slope (with 95% CI)	Intercept (with 95% CI)
Alanine	816	214	0.34 (0.31, 0.36)	0.44 (0.42, 0.46)	1.06 (0.98, 1.14)	-0.01 (-0.07, 0.04)
Glycerol	648	164	0.34 (0.32, 0.37)	0.43 (0.41, 0.44)	1.26 (1.16, 1.35)	-0.13 (-0.20, -0.07)
Glucose-cas	737	65	0.31 (0.28, 0.34)	0.42 (0.40, 0.44)	0.91 (0.83, 1.00)	0.09 (0.03, 0.15)

References

S1. Tiruvadi Krishnan, S., Männik, J., Kar, P., Lin, J., Amir, A., and Männik, J. (2021). Replication-related control over cell division in *Escherichia coli* is growth-rate dependent. bioRxiv, 10.1101/2021.02.18.431686 .
