# Supplementary material for: Distinguishing different modes of growth using single-cell data
Source: eLife. 2021 Dec 2;10:e72565. doi: 10.7554/eLife.72565 (PMC8727026; doi:10.7554/eLife.72565)
Supplement: Transparent reporting form [file elife-72565-transrepform1.docx]

***eLife’s* transparent reporting form**

We encourage authors to provide detailed information *within their submission* to facilitate the interpretation and replication of experiments. Authors can upload supporting documentation to indicate the use of appropriate reporting guidelines for health-related research (see [EQUATOR Network](http://www.equator-network.org/%20)), life science research (see the [BioSharing Information Resource](https://biosharing.org/)), or the [ARRIVE guidelines](http://www.plosbiology.org/article/info:doi/10.1371/journal.pbio.1000412) for reporting work involving animal research. Where applicable, authors should refer to any relevant reporting standards documents in this form.

If you have any questions, please consult our Journal Policies and/or contact us: [editorial@elifesciences.org](mailto:editorial@elifesciences.org).

**Sample-size estimation**

- You should state whether an appropriate sample size was computed when the study was being designed
- You should state the statistical method of sample size computation and any required assumptions
- If no explicit power analysis was used, you should describe how you decided what sample (replicate) size (number) to use

Please outline where this information can be found within the submission (e.g., sections or figure legends), or explain why this information doesn’t apply to your submission:

No sample size calculation was conducted for experimental samples. All data were originating from microfluidic experiments that involved a large number of cells. The experimental sample sizes used in analyzed data were for cells growing in M9 alanine (n=816), M9 glycerol (n=648), and M9 Glucose-cas (n=737). These are reported in Table S2 and figure legends where the data were used: Figure 2, Figure 2- figure supplement 1, Figure 4- figure supplement 1, and Figure 4- figure supplement 2.

**Replicates**

- You should report how often each experiment was performed
- You should include a definition of biological versus technical replication
- The data obtained should be provided and sufficient information should be provided to indicate the number of independent biological and/or technical replicates
- If you encountered any outliers, you should describe how these were handled
- Criteria for exclusion/inclusion of data should be clearly stated
- High-throughput sequence data should be uploaded before submission, with a private link for reviewers provided (these are available from both GEO and ArrayExpress)

Please outline where this information can be found within the submission (e.g., sections or figure legends), or explain why this information doesn’t apply to your submission:

Given that a large number of data points were used in the study, not all experiments were replicated. However, we ensured that all key variables (timing of the start of septum formation/onset constriction (T_n_), cell size (at birth, L_b_ and division, L_d_) and generation time (Td)) could not only be replicated but also replicated with similar means and SDs in several growth conditions and with different labels. We also verified that different fluorescent labels expressed from cells did not affect these quantities.

Cells that contained obvious errors in the image segmentation were excluded from the further analysis.

**Statistical reporting**

- Statistical analysis methods should be described and justified
- Raw data should be presented in figures whenever informative to do so (typically when N per group is less than 10)
- For each experiment, you should identify the statistical tests used, exact values of N, definitions of center, methods of multiple test correction, and dispersion and precision measures (e.g., mean, median, SD, SEM, confidence intervals; and, for the major substantive results, a measure of effect size (e.g., Pearson's r, Cohen's d)
- Report exact p-values wherever possible alongside the summary statistics and 95% confidence intervals. These should be reported for all key questions and not only when the p-value is less than 0.05.

Please outline where this information can be found within the submission (e.g., sections or figure legends), or explain why this information doesn’t apply to your submission:

Linear regression is used in the paper to obtain functional relationship between variables. The slope and the intercept of the best linear fit shown in Figures 2A-2B and Figure 2- figure supplement 1 and their 95% confidence intervals are presented in Table S2. The error bars for the binned data trend throughout the paper show the standard error (= standard deviation of the y-axis variable/$\surd N$, where N is number of points in that bin). This information is also provided in the relevant figure legends.

(For large datasets, or papers with a very large number of statistical tests, you may upload a single table file with tests, Ns, etc., with reference to sections in the manuscript.)

**Group allocation**

- Indicate how samples were allocated into experimental groups (in the case of clinical studies, please specify allocation to treatment method); if randomization was used, please also state if restricted randomization was applied
- Indicate if masking was used during group allocation, data collection and/or data analysis

Please outline where this information can be found within the submission (e.g., sections or figure legends), or explain why this information doesn’t apply to your submission:

Samples were not allocated to groups.

**Additional data files (“source data”)**

- We encourage you to upload relevant additional data files, such as numerical data that are represented as a graph in a figure, or as a summary table
- Where provided, these should be in the most useful format, and they can be uploaded as “Source data” files linked to a main figure or table
- Include model definition files including the full list of parameters used
- Include code used for data analysis (e.g., R, MatLab)
- Avoid stating that data files are “available upon request”

Please indicate the figures or tables for which source data files have been provided:

Experimental data used in the paper has been uploaded and is accessible through Dataverse, “Kar, Prathitha; Tiruvadi-Krishnan, Sriram; Männik, Jaana; Männik, Jaan; Amir, Ariel, 2021, "To bin or not to bin: analyzing single-cell growth data", https://doi.org/10.7910/DVN/BNQUDW, Harvard Dataverse, DRAFT VERSION”. The values of the model parameters used in the simulations are presented in Section 5.11 in the paper.
